# Functional Genomics of a Symbiotic Community: Shared Traits in the Olive Fruit Fly Gut Microbiota

**DOI:** 10.1093/gbe/evz258

**Published:** 2019-12-12

**Authors:** Frances Blow, Anastasia Gioti, Ian B Goodhead, Maria Kalyva, Anastasia Kampouraki, John Vontas, Alistair C Darby

**Affiliations:** 1 Institute of Integrative Biology, University of Liverpool, United Kingdom; 2 Department of Entomology, Cornell University, Ithaca, New York; 3 Bioinformatics Facility, Perrotis College, American Farm School, Thessaloniki, Greece; 4 School of Environment and Life Sciences, University of Salford, United Kingdom; 5 Department of Biology, University of Crete, Heraklion, Greece; 6 Institute of Molecular Biology & Biotechnology, Foundation for Research & Technology Hellas, Heraklion, Greece; 7 Pesticide Science, Agricultural University of Athens, Greece

**Keywords:** symbiosis, horizontal gene transfer, urease, adhesion, *Candidatus* Erwinia dacicola, *Tatumella* sp. TA1

## Abstract

The olive fruit fly *Bactrocera oleae* is a major pest of olives worldwide and houses a specialized gut microbiota dominated by the obligate symbiont “*Candidatus* Erwinia dacicola.” *Candidatus* Erwinia dacicola is thought to supplement dietary nitrogen to the host, with only indirect evidence for this hypothesis so far. Here, we sought to investigate the contribution of the symbiosis to insect fitness and explore the ecology of the insect gut. For this purpose, we examined the composition of bacterial communities associated with Cretan olive fruit fly populations, and inspected several genomes and one transcriptome assembly. We identified, and reconstructed the genome of, a novel component of the gut microbiota, *Tatumella* sp. TA1, which is stably associated with Mediterranean olive fruit fly populations. We also reconstructed a number of pathways related to nitrogen assimilation and interactions with the host. The results show that, despite variation in taxa composition of the gut microbial community, core functions related to the symbiosis are maintained. Functional redundancy between different microbial taxa was observed for genes involved in urea hydrolysis. The latter is encoded in the obligate symbiont genome by a conserved urease operon, likely acquired by horizontal gene transfer, based on phylogenetic evidence. A potential underlying mechanism is the action of mobile elements, especially abundant in the *Ca.* E. dacicola genome. This finding, along with the identification, in the studied genomes, of extracellular surface structure components that may mediate interactions within the gut community, suggest that ongoing and past genetic exchanges between microbes may have shaped the symbiosis.

## Introduction

Many insects house gut microbial communities that perform essential functions related to their diet or lifestyle (Dillon and Dillon 2004). Insect species with a dependence on a specialized gut microbial community for their fitness often have specialized alimentary structures to house microbes, and these microbes are also often vertically transmitted between mother and offspring to ensure the inoculation of subsequent generations (Salem et al. 2015). The gut microbiota of wild populations of the olive fruit fly *Bactrocera oleae* (Tephritidae) is numerically dominated by a single Gammaproteobacterium, “*Candidatus* Erwinia dacicola” (Capuzzo et al. 2005; Estes et al. 2009; Kounatidis et al. 2009; Estes et al. 2012; Ben-Yosef et al. 2015). The symbiont *Ca.* E. dacicola resides in the digestive tract throughout the insect’s lifecycle, in larvae within the midgut caeca and in adults in a specialized foregut diverticulum structure called the esophageal bulb, and is maternally transmitted between generations by egg smearing (Estes et al. 2009). *Candidatus* Erwinia dacicola has not been detected outside of the insect by environmental sampling, while efforts to culture it in vitro have so far been unsuccessful (Capuzzo et al. 2005). From the above, the bacterium qualifies as a specific obligate symbiont of *B. oleae*, but also a challenge to study experimentally. In this context, sequence data-based methods are of high value for the generation of hypotheses on the different roles of *Ca.* E. dacicola in host fitness.

In common with many holometabolous insects, the adult and juvenile stages of *B. oleae* feed on different food sources (Tzanakakis 2003), therefore the *Ca.* E. dacicola symbiosis is hypothesized to perform alternative functions during these developmental stages. In larvae, the symbiosis has been hypothesized to allow the insect to use ripening fruit by detoxifying defensive plant phenolic compounds, such as oleuropein (Soler-Rivas et al. 2000; Ben-Yosef et al. 2015), and sequestering amino acids for protein synthesis (Ben-Yosef et al. 2015). During adulthood, the native gut microbiota is thought to provision nitrogen to the host: Females harboring their native microbiota have a higher reproductive output on diets lacking essential amino acids (EAAs), and diets containing only nitrogen sources that are metabolically intractable to *B. oleae*, such as urea (Ben-Yosef et al. 2010, 2014). It is common for many insects to house microbes that increase the quantity or quality of dietary nitrogen by performing novel metabolic functions (Douglas 2009). For example, the intracellular symbiont *Blochmannia* within the bacteriocytes of *Camponotus* ants encodes urease that metabolizes dietary urea to ammonia from which it synthesizes essential and non-EAAs, subsequently transported to the hemolymph for host consumption (Feldhaar et al. 2007). A similar process is proposed to occur in *B. oleae* adults, which consume bird droppings containing ammonia and urea as part of their omnivorous diet (Ben-Yosef et al. 2014), but do not encode endogenous ureases (*B. oleae* genome accession number: GCF_001188975.1, Djambazian H, et al. 2018). However, the specific pathways and related enzymes involved in the metabolism and uptake of nitrogenous substrates remain to be elucidated in *B. olea*e.

Previous experimental approaches aiming to evaluate symbiotic function have tested the native microbial community of the olive fruit fly, which is dominated by, but not restricted to *Ca*. E. dacicola (Estes et al. 2009; Ben-Yosef et al. 2015; Blow, Vontas, et al. 2016; Blow et al. 2017; Koskinioti et al. 2019). In this study, we took advantage of recently available –omic resources for the symbiont (Blow, Gioti, et al. 2016, Pavlidi et al. 2017, Estes et al. 2018b) and further generated new sequence data from single-culture and metagenomic samples of the *B. oleae* gut community to: (1) investigate whether *Ca.* E. dacicola encodes the full gene repertoire required to provide dietary nitrogen to *B. oleae* and (2) identify other members of the *B. oleae* gut microbiota potentially capable of performing these functions, in order to investigate the functional significance of the previously observed variation in *B. oleae* community composition. For this purpose, we employed comparative genomic and phylogenetic analyses, considering the function of the gut community in the context of symbiosis.

## Materials and Methods

### Insect Material

*Bactrocera* *oleae* adults were obtained by collecting infested olives from trees in the grounds of the University of Crete (Heraklion, Greece) in October and November of 2014. Infested olives were suspended over sterile sand with a wire mesh, and third-instar larvae were allowed to emerge from the fruit. Flies pupated in the sand and were placed into Petri dishes in 10 cm^3^ plastic cages prior to emergence. Flies were maintained at 25 °C and 60% relative humidity and were supplied with artificial diet (19% hydrolyzed yeast, 75% icing sugar, 6% egg yolk). Each cage was provided with Milli-Q water in a clean plastic container and wax cones for oviposition.

### Preparation and Sequencing of 16S rRNA Gene Amplicon Libraries

DNA was extracted from a total of 52 whole *B. oleae* adults using the Qiagen DNeasy Blood and Tissue kit for Gram-positive bacteria (Qiagen, UK) following the manufacturer’s instructions. An additional bead-beating step using 3 mm carbide beads (Qiagen, UK) in a Qiagen tissue lyzer (Qiagen, UK) at 25 Hz for 30 s was employed. In order to assess the diversity and composition of the bacterial communities, bacterial 16S rRNA V4 regions were amplified by PCR using the universal primers F515 (5′-GTGCCAGCMGCCGCGGTAA-3′) and R806 (5′-GGACTACHVGGGTWTCTAAT-3′; Caporaso et al. 2011). Samples were dual-indexed for sequencing following the method in D’Amore et al. (2016). PCR reactions were performed in a total volume of 20 μl, containing 5 ng of template DNA, 10 μl NEBNext 2× High-Fidelity Master Mix (New England Biolabs), 0.3 μM of each primer, and 3.4 μl PCR-clean water. Thermal cycling conditions were 98 °C for 2 min, 10 cycles of 98 °C for 20 s, 60 °C for 15 s, and 70 °C for 30 s, with a final extension at 72 °C for 5 min. PCR products were purified with Agencourt AMPure XP beads (Beckman Coulter Genomics) and used as template for the second PCR reaction. Purified first-round PCR products were combined with 10 μl NEBNext 2× High-Fidelity Master Mix (New England Biolabs), and 0.3 μM of each barcoding primer containing adapters and indexes to a total volume of 20 μl. Thermal cycling conditions were 98 °C for 2 min, 15 cycles of 98 °C for 20 s, 55 °C for 15 s and 72 °C for 40 s, with a final extension at 72 °C for 60 s. PCR products were purified with Agencourt AMPure XP beads (Beckman Coulter Genomics) and quantified with the Qubit dsDNA High-Sensitivity assay (Life Technologies), and an Agilent Bioanalyzer High-Sensitivity DNA chip (Agilent). Samples were pooled at equimolar concentrations and size-selected in a range of 350–450 bp by Pippin-Prep (Sage Science). Sequencing was performed at the University of Liverpool Centre for Genomic Research on an Illumina MiSeq platform with V2 chemistry, generating 250 bp paired-end reads. All raw sequencing reads were deposited at NCBI under the BioProject accession PRJNA321174.

### Computational Analyses of 16S rRNA Sequencing Data

Raw sequencing reads were demultiplexed and converted to FASTQ format using CASAVA version 1.8 (Illumina 2011). Cutadapt version 1.2.1 (Martin 2011) was used to trim Illumina adapter sequences from FASTQ files. Reads were trimmed if 3 bp or more of the 3′ end of a read matched the adapter sequence. Sickle version 1.200 (Joshi and Fass 2011) was used to trim reads based on quality: Any reads with a window quality score of <20, or were <10 bp long after trimming, were discarded. BayesHammer was used to correct reads based on quality (Nikolenko et al. 2013). Paired-end reads were merged with a minimum overlap of 50 bp using PandaSeq (Masella et al. 2012). All subsequent analyses were conducted in QIIME version 1.8.0 (Caporaso, Kuczynski, et al. 2010). Sequences were clustered into Operational Taxonomic Units (OTUs) by de novo OTU picking with USEARCH (Edgar 2010). Chimeras were detected and omitted with UCHIME (Edgar et al. 2011) and the QIIME-compatible version of the SILVA 111 release database (Quast et al. 2012). The most abundant sequence was chosen as the representative for each OTU, and taxonomy was assigned to representative sequences by BLAST (Altschul et al. 1990) against the SILVA 111 release database, which was supplemented with several reference sequences for *Ca.* E. dacicola. OTUs were filtered from the data set if they matched the SILVA 111 database for chloroplasts or mitochondria. OTU representative sequences were aligned against the Greengenes core reference alignment (DeSantis et al. 2006) using PyNAST (Caporaso, Bittinger, et al. 2010). Previously published 16S rRNA gene amplicons from flies collected in Israel (Ben-Yosef et al. 2015) were employed for comparison with the above data. Since data generation methods for the samples from Israel varied slightly, all data analysis methods were standardized from read-error correction onwards in this study. *Tatumella* sp. TA1 prevalence was calculated as the proportion of individuals where *Tatumella* sp. TA1 16S rRNA was >0.01% of the total 16S rRNA gene copies from that sample, and relative abundance was the proportion of the total 16S rRNA gene copies.

### Isolation, Culture and Identification of *Tatumella* sp. TA1 from *B. oleae*

*Tatumella* sp. TA1 was identified from a two-day old male fly following the below isolation and culture procedures, all conducted under sterile conditions using aseptic technique or in a laminar flow cabinet. Individual two-day old adult *B. oleae* collected in Crete were surface-sterilized with 70% ethanol and rinsed twice in distilled water prior to homogenization with a plastic pestle in 20 μl nuclease-free water. 10 μl of the homogenate was spotted on to Columbia agar (Oxoid, UK) supplemented with 5% defibrinated horse blood (TCS Biosciences, UK), and plates were incubated at 25 °C for 48 h. Single colonies were picked and streaked onto Brain Heart Infusion (BHI) agar (Oxoid, UK) to establish pure cultures. Pure liquid cultures were generated by inoculating single colonies from BHI agar plates in to BHI liquid medium and incubating them at 25 °C for 48 h. Liquid cultures were cryopreserved following the addition of a cryoprotectant (20% [v/v] glycerol final concentration) and storage at –80 °C. In order to identify isolates, single colonies from BHI agar plates were picked and inoculated into 10 μl nuclease-free water in a PCR tube. Tubes were incubated at 95 °C for 5 min to lyse cells and isolate DNA. A 1500 bp region of the 16S rRNA gene was amplified with universal primers 8F (5′-AGAGTTTGATCMTGGCTCAG-3′) and 1492R (5′-CCCCTACGGTTACCTTGTTACGAC-3′). Reactions were performed in a total volume of 25 μl containing 12.5 μl 2× MyTaq Red (Bioline, UK), 0.5 μl of each 10 μM primer stock, 1 μl template DNA, and 10.5 μl nuclease-free water. Thermal cycling conditions were 95 °C for 5 min, 30 cycles of 95 °C for 30 s, 56 °C for 45 s, 72 °C for 90 s, and a final extension at 72 °C for 7 min. PCR products were Sanger sequenced with forward primer 8F by GATC (GATC, Cologne) and resulting sequences were subjected to BLAST analysis (Altschul et al. 1990) against the GenBank database (http://www.ncbi.nlm.nih.gov/; last accessed April 25, 2016) to allow taxonomic identification by similarity.

### Library Preparation and Sequencing of the *Tatumella* sp. TA1 Genome

DNA from *Tatumella* sp. TA1 was extracted as follows: Cryopreserved isolates were revived by streaking on to BHI agar and incubated at 25 °C for 72 h. Single colonies were inoculated into BHI broth and incubated at 25 °C until cultures reached an OD600 of 0.3. Cultures were pelleted by centrifugation at 6,000 × g for 6 min. The supernatant was removed, and cells were resuspended in DNA elution buffer at a concentration of 1 × 10^5^ Colony Forming Units (CFUs) ml^−1^. DNA was extracted using the Zymo Quick DNA Universal Kit (Zymo, UK) following the manufacturer's instructions for biological fluids and cells with the following amendments to the protocol: Samples were incubated with proteinase K at 55 °C for 30 min rather than 10 min. DNA was purified with Ampure beads (Agencourt) at a 1:1 ratio, and stored at 4 °C until library preparation. DNA was sheared to 10 kb using Covaris G-tubes following the manufacturer’s guidelines, and library preparation was performed with the SMRTbell library preparation kit (Pacific Biosciences) following the manufacturer’s instructions. The Qubit dsDNA HS assay (Life Technologies, UK) was used to quantify the library, and the average fragment size was determined using the Agilent Bioanalyzer HS assay (Agilent). Size selection was performed with the Blue Pippin Prep (Sage Science) using a 0.75% agarose cassette and the S1 marker. The final SMRT bell was purified with Agencourt AMPure XP beads (Beckman Coulter Genomics) and quantified with the Qubit dsDNA High-Sensitivity assay (Life Technologies), and an Agilent Bioanalyzer High-Sensitivity DNA chip (Agilent). The SMRTbell library was annealed to sequencing primers at values predetermined by the Binding Calculator (Pacific Biosciences), and sequencing was performed on two SMRT cells using 360-min movie times. Pacific Biosciences sequencing and library preparation of the *Tatumella* sp. TA1 isolate was performed at the University of Liverpool Centre for Genomic Research on a Pacific Biosciences RS II sequencer.

### Genome Assembly and Annotation

The *Tatumella* sp. TA1 draft genome was assembled from 365,445 PacBio subreads with a mean length of 6,926 bp using the Hierarchical Genome Assembly Process (HGAP) workflow (Chin et al. 2013). It is available at NCBI under the accession numbers CP033727–CP033728. The HGAP pipeline comprised pre-assembly error correction of subreads based on read length and quality, assembly with Celera, and assembly polishing with Quiver.

The assembled genome and plasmid were annotated with PROKKA version 1.5.2 (Seemann 2014). To further investigate the extracellular membrane structures and mobile elements encoded in the *Ca.* E. dacicola, *Tatumella* sp. TA1 and *Enterobacter* sp. OLF assemblies, genomes were re-annotated with RAST (Overbeek et al. 2014). These annotations are available in supplementary table S1, Supplementary Material online (A–E).

To determine whether the pTA1 plasmid from *Tatumella* sp. TA1 was present in *Ca.* E. dacicola, we also assembled the plasmid from reads used to generate the *Ca.* E. dacicola Oroville assembly, which did not contain *Tatumella* sp. TA1 chromosomal DNA (SRA accession number SRP155530, Estes et al. 2018b). To assemble the pTA1 plasmid, reads were trimmed with Trimmomatic version 0.39 (Bolger et al. 2014) and mapped to the *Tatumella* sp. TA1 chromosomal and plasmid PacBio assemblies using Bowtie2 (Langmead et al. 2009). Read mapping coverage was estimated using the -depth function in Samtools (Li et al. 2009) using a mapping quality cutoff of 30.

### Phylogenetic Analyses

For the species tree, allowing phylogenetic placement of *Tatumella* sp. TA1 and *Ca.* E. dacicola, taxa were selected based on (Palmer et al. 2017), which presents a robust phylogeny of the *Erwinia*, *Pantoea*, and *Tatumella* genera of *Erwiniaceae* using genome-wide orthologs. Where Palmer et al. (2017) included several conspecific taxa, we chose the most biologically relevant of the identical taxa. The Western Flower Thrip (WFT)-associated bacteria BFo1 and BFo2 were included in order to validate phylogenetic similarities with *Ca.* E. dacicola and *Tatumella* TA1 based on 16S rRNA gene sequences. We also included several symbiotic taxa identified in Chen et al. (2017) as associated with *Orius*, which we chose based on their phylogenetic placement: OLMDLW33, phylogenetically close to BFo1, OPLPL6, close to BFo2, as well as OLMTSP26 and OLMTSP33, two representatives of the *Erwiniaceae*-like clade (preliminary phylogenetic analyses showed that most sequences were identical among the seven taxa). A full list of the 50 genomes employed for this analysis can be found in supplementary table S2*A*, Supplementary Material online, including the seven outgroup taxa and the three available *Ca.* E. dacicola draft genome assemblies (ErwSC, IL, and Oroville). Genomes were annotated with PROKKA version 1.5.2 using the default settings, and 287 single-copy orthologs present in all taxa were identified using OrthoMCL version 1.4 with default parameters (Li et al. 2003). The “reference” tree for the urease phylogenetic analysis was estimated from eight genes randomly selected from the set of 287 single-copy orthologs, with a total of 5,461 informative sites; we assumed neutral evolution status for these genes based on their predicted housekeeping functions (supplementary table S2*A*, Supplementary Material online). For the urease subunit alpha (*ureC*) gene tree, homologs were retrieved based on BlastP queries against the nr database at NCBI (restricted to taxid = Bacteria). We included as many taxa common to the reference and species tree as possible, to ensure meaningful comparisons. Additional homologs were retrieved by targeted searches in RefSeq (full list of species names and accession numbers in supplementary table S2*B*, Supplementary Material online). For all trees, amino acid sequences were aligned with MUSCLE version 3.8.31 (Edgar 2004), and informative sites were selected with Gblocks version 091b (Castresana 2000). Maximum-Likelihood trees were estimated using IQ-TREE version 1.6.0 after automated model selection (Nguyen et al. 2015), with 300 random trees as burn-in. Node support was calculated using 1,000 ultra-fast bootstraps (Minh et al. 2013). The multi-locus coalescent-based species and reference trees were estimated from individual ortholog trees using Astral III, with node support calculated using 1,000 bootstraps (Zhang et al. 2018). Trees were rooted and visualized using FigTree v.1.4.0 (http://tree.bio.ed.ac.uk/software/figtree/).

### Statistical Analyses

All statistical analyses were performed in R version 3.3.3 (R Core Team 2017). Comparisons of GC content mean ranks, means and distributions were performed with Mann–Whitney, Welch’s *t*-test (allowing for comparison of unequal variance-samples) and Kolmogorov–Smirnov tests. For these comparisons, the GC content of the urease operon and the whole genome of *Ca.* E. dacicola were calculated either using overlapping 50 bp windows or, for GC3, GC1, and GC2 at the corresponding positions of predicted coding genes (CDS).

### Data Availability

16S rRNA gene amplicon sequencing data are available as part of NCBI BioProject PRJNA321174. The *Tatumella* sp. TA1 genome is available at NCBI under accession numbers CP033727 (chromosome) and CP033728 (plasmid pTA1). Alignments for figures 2 and 4 are available upon request from the corresponding authors. Details of previously published *Ca.* E. dacicola genomes are available in supplementary table S2*A*, Supplementary Material online, and RAST annotations and sequences of the *Ca.* E. dacicola transcriptomic data set employed from Pavlidi et al. (2017) are available in supplementary table S1*F*, Supplementary Material online.

## Results and Discussion

### A Novel Member of the Gut Microbiota Associated with Mediterranean Populations of *B. oleae*

**Fig. 1.**
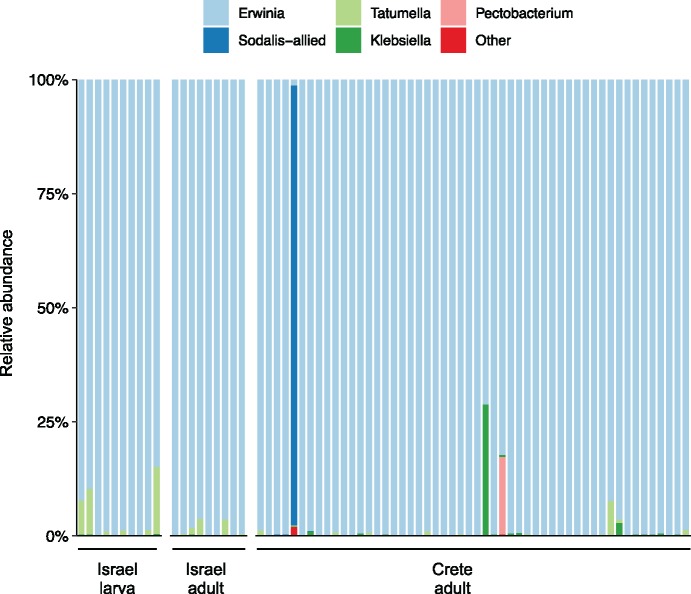
—Relative abundances of bacterial genera associated with individual *Bactrocera oleae* collected in Israel and Crete. Each bar represents the bacterial community associated with an individual insect, as assessed by *16S rRNA* gene amplicon sequencing of whole wandering larvae from Israel (Israel larva, *n* = 10), adults from Israel (Israel adult, *n* = 9), and adults from Crete (Crete adult, *n* = 52). *16S rRNA* gene amplicon sequencing data for individuals collected in Israel were previously published in Ben-Yosef et al. (2015) and reanalyzed for the purposes of this study. The category “Other” represents low abundance bacterial genera and reached a maximum value of 2% in one individual, and otherwise ranged between 0.1% and 0.3% of *16S rRNA* gene amplicon sequences per individual.

To explore the ecology of the *B. oleae* gut, we examined its bacterial community composition by 16S rRNA gene amplicon sequencing of samples from olive fruit fly populations collected in Crete. This approach, combined with culturing of selected isolates, allowed identifying and isolating into axenic culture a culture-viable member of the gut microbiota that has not been characterized before, referred to here as *Tatumella* sp. TA1. Reanalysis of 16S rRNA gene data from a previous study demonstrated that *Tatumella* sp. TA1 is also present in wild populations from Israel (Ben-Yosef et al. 2015; fig. 1). We detected *Tatumella* sp. TA1 in 88.9–90% of individuals from populations in Israel, in both adults and larvae, and in 26.9% of individuals from populations in Crete (fig. 1). Notably, *Tatumella* sp. TA1 was not detected in any of the culture-independent analyses of the gut microbiota of US olive fruit flies from two distinct geographic regions, though these data were not suitable for reanalysis in this study as they were singly-cloned 16S rRNA gene amplicons (Estes et al. 2009, 2012), and so could not be compared with the community-wide 16S rRNA gene amplicon studies. Similarly, *Enterobacter* sp. OLF, a member of the gut community identified in US populations of *B. oleae* (Estes et al. 2009), was not detected in either of the Mediterranean populations studied here. Collectively, these results suggest that *Tatumella* sp. TA1 and *Enterobacter* sp. OLF are facultative members of the gut community and geographically restricted to US and Mediterranean populations of the olive fruit fly, respectively.

In addition to *Tatumella* sp. TA1, 16S rRNA gene diversity analyses identified three other bacterial genera (all members of the family *Enterobacteriaceae*) as frequently associated with *B. oleae* and sometimes present at high relative abundances: *Pectobacterium*, *Klebsiella*, and a *Sodalis*-allied bacterium (Snyder et al. 2011; fig. 1). For example, 97% of the 16S rRNA gene sequences from one adult fly from Crete originated from a *Sodalis-*allied bacterium, with *Ca.* E. dacicola 16S rRNA comprising just 1.3% of the sequenced 16S rRNA genes in this individual (fig. 1). It is not uncommon for some individuals in the population to house bacterial communities dominated by taxa other than *Ca.* E. dacicola (Estes et al. 2012; Ben-Yosef et al. 2014; Koskinioti et al. 2019), or that *Ca.* E. dacicola may even be absent from some individuals (Kounatidis et al. 2009; Estes et al. 2012, 2014). Of the other taxa that colonize *B. oleae*, *Tatumella* sp. TA1 was the most frequent and the most abundant bacterial taxon after *Ca.* E. dacicola in *B. oleae* larvae (fig. 1), indicating that *Tatumella* sp. TA1 may be vertically transmitted from mother to offspring, or readily acquired from the environment by *B. oleae* larvae. We therefore chose to perform a genomic comparison of *Tatumella* sp. TA1 and *Ca.* E. dacicola to determine whether functional redundancy with the obligate symbiont *Ca.* E. dacicola may enable *Tatumella* sp. TA1 to colonize and exploit the *B. oleae* gut environment.

We used PacBio RS II sequencing to generate a draft genome sequence for *Tatumella* sp. TA1. The assembly comprised a chromosomal sequence of 3,389,139 bp and a circular plasmid sequence of 49,211 bp, named pTA1 (total genome size 3.4 Mb). Read coverage of both the chromosome and pTA1 was ∼800× and both the chromosome and plasmid sequences have been circularized. The full *Tatumella* sp. TA1 genome encodes a total of 3,309 protein coding genes, 72 tRNAs, and 22 rRNAs. The pTA1 plasmid had a total of 69 annotated genes (supplementary table S1*D*, Supplementary Material online), including Tra and Trb operons for conjugative plasmid transfer, and ccdAB and hicAB and toxin–antitoxin cassettes, which enable plasmid transfer and persistence in other systems, respectively (Wilkins and Lanka 1993; Syed and Lévesque 2012). The GC content of the *Tatumella* sp. TA1 genome is 48.6%, and both this and the genome size are comparable to those of previously published genomes of *Tatumella* species, many of which are host-associated (Hollis et al. 1981; Marín-Cevada et al. 2010; Chandler et al. 2014). The genome assembly is predicted to be 100% complete following the method used in Rinke et al. (2013), which detected 138/138 single-copy marker genes.

### Phylogenetic Placement of Obligate and Facultative Members of the *B. oleae* Gut Microbiota

**Fig. 2.**
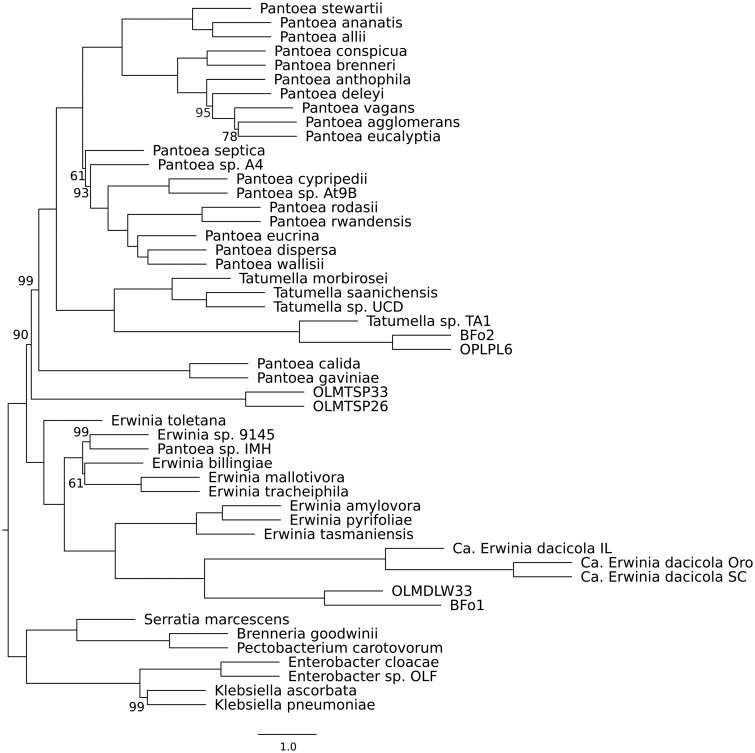
—Maximum-Likelihood tree estimated from amino acid alignments of 287 single-copy orthologs retrieved from whole genome assemblies of 41 *Erwinia, Pantoea*, and *Tatumella*, 2 *Erwiniaceae*, and 7 outgroup taxa. Full names and accession numbers of the genes used are available in supplementary table S2*A*, Supplementary Material online. Numbers on nodes correspond to bootstrap support (only values <100 are shown).

To resolve the phylogenetic relationships among the newly identified taxa in our and recent studies (Facey et al. 2015; Chen et al. 2017), we performed a phylogenomic analysis of 287 single-copy orthologs from a total of 50 bacterial taxa, including 41 taxa from the genera *Erwinia*, *Pantoea*, and *Tatumella*, along with two members of the *Erwiniaceae*, and seven outgroup taxa (full list in supplementary table S2*A*, Supplementary Material online). In agreement with the most recent comprehensive phylogeny of the *Erwiniaceae* (Palmer et al. 2017), the phylogenomic analysis supports the phylogenetic placement of *Tatumella* sp. TA1 within the *Tatumella* genus (fig. 2). It further confirms that *Tatumella* sp. TA1 and *Enterobacter* sp. OLF are distinct members of the order *Enterobacterales*, indicating that they are unique components of the *B. oleae* gut microbiota. In addition, the phylogeny showed that, despite an evolutionary association with *B. oleae* and vertical transmission, which are expected to change genome characteristics through altered selection pressures and drift (McCutcheon and Moran 2011), *Ca.* E. dacicola clusters within the *Erwinia* genus (fig. 2).

The closest phylogenetic relatives of *Tatumella* sp. TA1 and *Ca.* E. dacicola are the thrip symbionts BFo2 and BFo1, respectively (Facey et al. 2015). The clustering of these taxa may represent comparable lifestyles. In a result analogous to diet experiments in olive flies (Ben-Yosef et al. 2010, 2014), the fitness benefits of the WFT gut-lumen symbiont BFo1 were shown to be condition-dependent and only apparent on diets lacking a balanced source of amino acids, when gut bacteria are presumed to synthesize and provision adult WFT with amino acids required for protein synthesis (de Vries et al. 2004). Besides, both BFo1 and BFo2 also inhabit the gut lumen and are vertically transmitted between generations by an extracellular route (de Vries et al. 2001), as has been demonstrated previously for *Ca.* E. dacicola (Sacchetti et al. 2008; Estes et al. 2009). Interestingly, members of the genus *Orius*, which are omnivorous Anthrocorids and predators of the WFT in the Mediterranean (Bosco et al. 2008; Mouden et al. 2017), also harbor BFo1 and BFo2-like culturable symbionts named OLMDLW33 and OPLPL6, respectively (Chen et al. 2017); the current phylogenomic analysis demonstrates that these taxa most probably belong to the *Erwinia* and *Tatumella* genus, respectively. The co-occurrence of *Erwinia* and *Tatumella*-allied taxa in these three phylogenetically distinct insect species (olive fruit fly, WFT, *Orius*), merits further investigation to elucidate whether specific traits, of both the host insect and its bacterial partners, may predispose them to co-associations.

### Functional Redundancy of Nitrogen Assimilation Genes in Members of the *B. oleae* Gut Microbiota

**Fig. 3.**
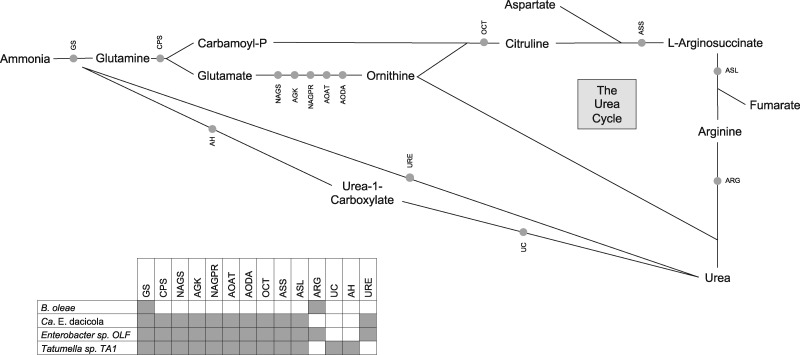
—Proposed pathways for urea hydrolysis and recycling of nitrogen into host and microbial metabolic pathways via ammonia, glutamine and glutamate. The genes encoding the illustrated enzymes were detected in genome assemblies of *Candidatus* Erwinia dacicola (Blow, Gioti, et al. 2016), *Tatumella* sp. TA1 (this study), both annotated with PROKKA, and *Enterobacter* sp. OLF annotated as described in Estes et al. (2018a). GS (Glutamine synthetase E.C. 6.3.1.2); CPS (Carbamoyl-phosphate synthase E.C. 6.3.4.16; E.C. 6.3.5.5); NAGS (N-acetylglutamate synthase E.C. 2.3.1.1); AGK (Acetylglutamate kinase E.C. 2.7.2.8); NAGPR (N-acetyl-gamma-glutamyl-phosphate reductase E.C. 1.2.1.38); AOAT (Acetylornithine/succinyldiaminopimelate aminotransferase E.C. 2.6.1.11); AODA (Acetylornithine deacetylase E.C. 3.5.1.16); OCT (Ornithine carbamoyltransferase E.C. 2.1.3.3); ASS (Argininosuccinate synthase E.C. 6.3.4.5); ASL (Argininosuccinate lyase E.C. 4.3.2.1); ARG (Arginase E.C. 3.5.3.1); UC (Urea carboxylase E.C. 6.3.4.6); AH (Allophanate hydrolase E.C. 3.5.1.54); URE (Urease E.C. 3.5.1.5).

We investigated the hypothesis that members of the *B. oleae* gut microbiota supplement the olive fruit fly diet with nitrogen by focusing on both obligate and facultative bacterial members. The annotated genomes of the obligate *Ca.* E. dacicola (Blow, Gioti, et al. 2016) and facultative *Tatumella* sp. TA1 (this study) and *Enterobacter* sp. OLF (Estes et al. 2018a) all encode enzymes that metabolize urea to ammonia and carbon dioxide (fig. 3, supplementary table S3*A*, Supplementary Material online): *Ca.* E. dacicola and *Enterobacter* sp. OLF encode ureases (EC 3.5.1.5), and *Tatumella* sp. TA1 encodes urea carboxylase (EC 6.3.4.6) and allophanate hydrolase (EC 3.5.1.54). None of these enzymes is encoded in the genome of *B. oleae* (supplementary table S3*A*, Supplementary Material online). A BlastP and TBlastN search of the *Ca.* E. dacicola transcriptome data (supplementary table S1*F*, Supplementary Material online) from larvae developing in green and black olives (Pavlidi et al. 2017) did not identify any of the urease genes, indicating that urease may not be expressed at the larval stage in the obligate symbiont. This result is expected, since at this stage there is no dietary source of urea. Therefore, a hypothesis to further explore is that urease expression in *Ca.* E. dacicola is inducible by host or dietary cues present during *B. oleae* adulthood. Similarly, the closest homologs to the *Ca.* E. dacicola urease gene (see next section) come from two plant pathogens, *Gibbsiella quercinecans* and *Brenneria goodwinii*, previously characterized as negative for urease function (Brady et al. 2010; Denman et al. 2012), which indicates that urease function may be inducible under specific conditions.

In addition to urea hydrolysis, *Ca.* E. dacicola, *Tatumella* sp. TA1 and *Enterobacter* sp. OLF all encode biosynthetic operons for nonessential and EAAs, with further evidence for expression during larval feeding in *Ca.* E. dacicola (supplementary table S3*B*, Supplementary Material online, transcript sequences available in supplementary table S1*F*, Supplementary Material online). Glutamine synthetase (EC 6.3.1.2) is also present in all three taxa and expressed in the *Ca.* E. dacicola transcriptome data. This enzyme, also encoded by the host genome (RefSeq accession GCF_001188975.1), incorporates nitrogen from ammonia into the non-EAA glutamine (fig. 3), which can be channeled into downstream metabolic processes including EAA biosynthesis (Feldhaar et al. 2007; Sabree et al. 2009).

We also investigated the hypothesis that host nitrogenous waste, which would otherwise be excreted, is the source of nitrogen assimilated to non-EAAs by the gut microbiota. Urea could come from recycling of host waste uric acid (Ben-Yosef et al. 2014), as has been observed in other insect symbionts that upgrade the nitrogen content of the host diet (Potrikus and Breznak 1981; Sasaki et al. 1996; Kashima et al. 2006; Feldhaar et al. 2007; Sabree et al. 2009). We found no genomic evidence that urea can be produced via uricolysis in either the host or any member of the so-far identified and sequenced microbiota: BlastP searches against shotgun sequencing data of olive fruit fly gut samples and corresponding metagenomic assemblies of the gut microbial community (NCBI BioProject accession PRJNA326914) failed to identify the required enzyme allantoicase (EC 3.5.3.4; , supplementary fig. S1, Supplementary Material online, supplementary table S3*A*, Supplementary Material online). However, *B. oleae* does encode an arginase (EC 3.5.3.1), which can hydrolyze the EAA arginine to urea and ornithine and could provide urease-degrading bacteria with a supply of urea (fig. 3). One hypothesis would be that *B. oleae* might enrich its gut microbiota with ureolytic bacteria such as *Ca.* E. dacicola through inducible delivery of urea produced by arginase activity. Signal peptides were not detected when individual urease proteins or the full operon from *Ca.* E. dacicola were analyzed with SignalP version 4.0 (Petersen et al. 2011), indicating that, if expressed, the urease encoded by *Ca.* E. dacicola is intracellular. This suggests that specific uptake of urea and excretion of its hydrolysis products may either be coordinated with the host, or that microbial cells are lysed after the hydrolysis of urea, in order for the host to gain the observed nutritional benefit when urea is added to the diet (Ben-Yosef et al. 2010, 2014). A key component of future studies should be to track the fate of metabolites resulting from urea hydrolysis: Whether they are retained by microbes for endogenous metabolism, or whether they are trafficked back to the host as, for example, ammonia or glutamine.

### Evidence for Horizontal Gene Transfer of the Urease Operon in the Obligate Symbiont *Ca.* E. dacicola

The *Ca.* E. dacicola urease enzyme is encoded, as in all ureolytic bacteria, by a cluster of eight adjacent genes arranged in an operon and denoted as *ureDABCEJFG*, with *ureABC* encoding structural proteins of the enzymatic complex, *ureDEFG* accessory proteins, and *ureJ* the transcriptional regulator. The urease operon appears incomplete at its 3′ end in the ErwSC assembly (Blow, Gioti, et al. 2016), missing *ureG*. However, *ureG* is present in the *Ca.* E. dacicola genome, as confirmed by BlastP queries against the initial set of metagenomic reads obtained from shotgun sequencing (NCBI BioProject accession PRJNA326914). The urease operon is also present in two additional *Ca.* E. dacicola assemblies (IL and Oroville) reconstructed with different methods and from different olive fly populations (supplementary table S2*A*, Supplementary Material online), and is complete (5,962 bp total length) in these assemblies, including *ureG*. The three “versions” of the operon from different assemblies are 99% similar at the nucleotide level for the genes present. One explanation for the absence of *ureG* in the ErwSC assembly is overtrimming, aiming to minimize the risk of including non*Ca.* E. dacicola scaffolds, since metagenomic sequencing data included DNA from several species, such as *Tatumella* sp. TA1. The differences between the reduced ErwSC and the more complete Oroville assembly are outlined in detail in Estes, Hearn, Agrawal, et al. (2018). In any case, the presence and similarity of the operon in all three *Ca.* E. dacicola assemblies despite their differences and despite the presence of different facultative taxa in the gut microbiome of *B. oleae* from distinct geographic populations, indicate that the urease operon is highly conserved in *Ca.* E. dacicola.

**Fig. 4.**
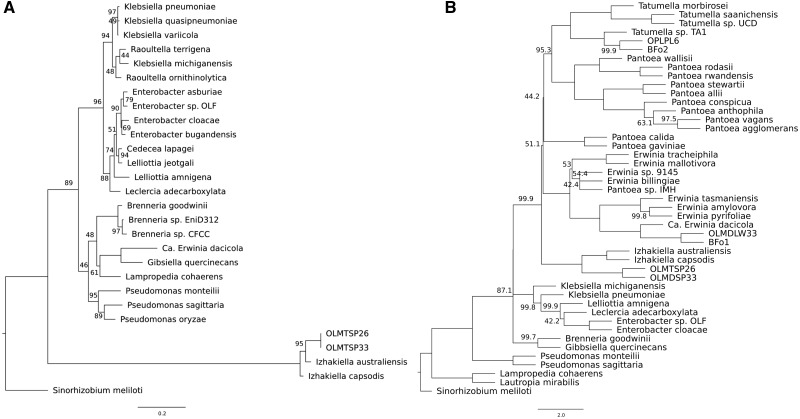
—Maximum-Likelihood trees estimated from amino acid alignments of (*A*) the urease subunit alpha (*ureC*) gene and (*B*) eight single-copy orthologs, representing a “neutral reference.” Rooting of both trees was performed with the most phylogenetically remote taxon, the Alphaproteobacterium *Sinorhizobium meliloti*. Full names and accession numbers for the genes (*A*) and genomes (*B*) used are available in supplementary tables S2*B* and S2*A*, Supplementary Material online, respectively. Numbers on nodes correspond to bootstrap support (only values <100 are shown).

In contrast to, for example, the *Tatumella* sp. TA1 urea carboxylase and allophanate hydrolase that have orthologs in other *Tatumella* species, *Ca.* E. dacicola is the only species of the genus *Erwinia* that encodes urease genes, to the best of our knowledge. Among the recently identified *Erwiniaceae* symbiotic taxa isolated from *Orius* insects (Chen et al. 2017), seven encode urease genes. However, a phylogenomic analysis including orthologs from two representatives of these nearly identical taxa (OLMTSP33 and OLMTSP33) showed that they do not belong to the genus *Erwinia*, but represent an unknown *Erwiniaceae* genus (fig. 2). Moreover, the most closely related taxon to *Ca.* E. dacicola (OLMDLW33, fig. 2) does not encode urease (but most probably has the ability to hydrolyze urea through the urea carboxylase and allophanate hydrolase that it encodes). Examination of the phylogeny of *ureC*, the longest structural gene of the operon, provided evidence for the acquisition of the urease operon in *Ca.* E. dacicola by horizontal gene transfer (HGT): The *Ca.* E. dacicola *ureC* history (fig. 4*A*), is different from that of the species history, as shown by the comparison to a “reference” tree, reconstructed from eight neutral single-copy orthologs (fig. 4*B*). The reference tree accurately depicts the known phylogenetic relationships between *Ca.* E. dacicola and other Proteobacteria, in agreement with the recently published comprehensive phylogeny (Palmer et al. 2017) and the species tree presented in this study (fig. 2). In contrast, the *Ca.* E. dacicola *ureC* protein groups together with proteins from more distant Gammaproteobacteria of the *Enterobacterales* order (*G. quercinecans*, *B. goodwinii*), and a distantly related Betaproteobacterium (*Lampropedia cohaerens*), and not with the *Erwiniaceae* taxa OLMTSP33 and OLMTSP26 from *Orius*, which form a well-supported outgroup to all of the above (fig. 4*A*). This latter observation indicates potential convergent evolution within the *Erwiniaceae* in symbionts of two different insect systems.

Hacker and Kaper (2000) defined genomic islands (GEIs) in bacteria as small (<10 kbp), syntenic blocks of genes acquired by HGT. It has been observed that GEIs share some common characteristics, such as the encoding of genes offering a selective advantage to the host, flanking by mobile genetic elements (MGEs) such as transposases, insertion close to tRNA genes, and a GC content different from that of the rest of the genome (Juhas et al. 2009). The ureolytic capacity conferred to *Ca.* E. dacicola by the urease operon is potentially an example of acquired selective advantage, since it increases the availability of nutrients to the symbiont itself or to the host, upon which it is dependent. Thus, it is tempting to argue that the urease operon represents a GEI in *Ca.* E. dacicola. Similarly to other GEIs, it has a distinct (lower) GC content compared with the rest of the genome (mean GC_urease_ = 50.2, mean GC_genome_ = 53.5), and this difference is statistically significant (*P*_Mann__–__Whitney_ < 0.00001, *P*_Welch_ < 0.00001). A statistically significant lower GC content was also observed when only the variable third codon position of all urease genes of the operon versus all predicted CDS of *Ca.* E. dacicola were compared (mean GC3_urease_ = 45.7, mean GC3_all-CDS_ = 59.7, *P*_Mann__–__Whitney_ < 0.001, *P*_Welch_ < 0.0001). In contrast, the first and second codon positions were not significantly different between urease genes and the rest of CDS of *Ca.* E. dacicola, indicating that the differences in GC content are driven by the third codon position. Moreover, the distribution of both GC and GC3 values sampled from the urease operon is significantly different from the whole-genome distribution (*P*_Kolmogorov_ < 0.0001 in both tests). Unfortunately, proximity of the operon to MGEs and tRNAs (another “signature” of GEIs) could not be confirmed in *Ca.* E. dacicola, since its genomic architecture remains unresolved: The operon appears in a separate scaffold in all three available assemblies (e.g., IL assembly accession number: LJAM02000124.1), none of which has been further extended and ordered with the use of long insert-size libraries. MGEs are frequently associated with fragmented assemblies, and in the ErwSC assembly, we detected a transposase fragment upstream of the first gene of the operon, *ureD*, in the scaffold containing the urease. However, mate-pair library sequencing data from Cretan olive fruit fly guts (accession number: SRX1896451) did not support the presence of the transposase upstream of *ureD* and did not allow identification of the operon’s flanking scaffolds.

The present data do not allow us to identify the date of the acquisition of the urease operon, nor its taxonomic origin; a further complication is that HGT events commonly occur recurrently among bacterial lineages. The observation that the GC content of the urease operon is distinct from that of its host may argue in favor of a relatively recent acquisition, as previously proposed for a different *Ca.* E. dacicola GEI (Estes, Hearn, Agrawal, et al. 2018), but one cannot conclude without dating the symbiosis. Similarly, regarding the donor, the low bootstrap support for the clades that link the groups comprising *Ca.* E. dacicola, *L. cohaerens*, and *Brenneria* to the rest of the clades indicates that the original donor of the urease cluster might be extinct or not yet sequenced. One possibility is that it is a free-living bacterium encountered by *Ca.* E. dacicola before its transition to obligacy with *B. oleae* or a yet-uncharacterized gut bacterium. In line with the latter, there is abundant evidence that members of gut microbial communities in vertebrates and invertebrates undergo HGT, and that this can subsequently influence functional traits (Petridis et al. 2006; Smillie et al. 2011; Stecher et al. 2012; Baker et al. 2018).

### Exchanges of Genetic Material between Members of the *B. oleae* Gut Microbiota

HGT events are commonly driven by MGEs, we thus searched the annotated symbiont genomes for such elements. MGEs, including transposases, repetitive DNA regions and phages, are abundant in all three *Ca.* E. dacicola assemblies, representing an average 34% of the obligate symbiont’s genome, in contrast to the distinctively lower MGE content of the *Tatumella* sp. TA1 and *Enterobacter* sp. OLF genomes (table 1). However, these percentages should not to be taken at absolute value due to the inherent difficulties in computational annotation of MGEs, especially from metagenomic samples (Jørgensen et al. 2014), while they might represent an overestimate due to the draft—and often fragmented—nature of the assemblies. Still, this result is in line with observations of high transposase content in bacteria that recently adopted a symbiotic lifestyle (Gil et al. 2008; Ran et al. 2010), and expression of MGEs in *B. oleae* larvae feeding on green and black olives (Pavlidi et al. 2017). These findings overall support the idea that genetic exchange between *Ca.* E. dacicola and members of the *B. oleae* gut microbial community may occur.

**Table 1:** Abundance of Different Categories of Mobile Genetic Elements (MGEs) in the *Candidatus* Erwinia dacicola (Three Currently Available Assemblies), *Tatumella* sp. TA1, and *Enterobacter* sp. OLF Genomes, as Annotated by RAST Subsystems

Genome	Transposase	Mobile Element	Insertion Element	Repeat Region	Phage & Phage-associated	Total Predicted MGEs	Total Predicted CDS	% MGEs of Total CDS
*Ca.* E. dacicola Oroville	134	196	16	637	335	1,318	4,900	26.90
*Ca.* E. dacicola SC	208	439	29	1,016	74	1,766	4,184	42.21
*Ca.* E. dacicola IL	191	313	26	700	225	1,455	4,219	34.49
*Tatumella* sp. TA1	19	23	0	42	66	150	3,575	4.20
*Enterobacter* sp. OLF	14	11	0	87	70	182	5,001	3.64

An example of genetic material exchange within the gut symbiotic community possibly concerns plasmid pTA1: The plasmid, reconstructed as part of the genome of *Tatumella* sp. TA1, isolated in the present study from Mediterranean populations of *B. oleae* (GenBank accession number: CP033728), was also detected in the *Ca.* E. dacicola Oroville assembly, which was generated from *B. oleae* populations from the US, where *Tatumella* sp. TA1 has not yet been detected (Estes et al. 2012). We were able to successfully assemble the plasmid sequence from raw reads used in the *Ca.* E. dacicola Oroville assembly, and alignment of pTA1 assembled from *Tatumella* sp. TA1 and *Ca.* E. dacicola Oroville raw reads indicates that the plasmid is shared by these taxonomically distinct and geographically isolated bacterial taxa (supplementary fig. S2, Supplementary Material online). To confirm that the *Tatumella* sp. TA1 plasmid and not the chromosome was present in the community of bacterial cells sequenced to generate the *Ca.* E. dacicola Oroville assembly, we adopted two approaches: Firstly, we used BlastN to search the contigs from the Oroville assembly to identify 16S rRNA gene sequences from taxa other than *Ca.* E. dacicola, and in line with previous analyses by the authors of the study (Estes et al. 2018b) did not find any. Secondly, we mapped the reads used to generate the *Ca.* E. dacicola Oroville assembly (SRA accession SRP155530) to the *Tatumella* sp. TA1 chromosome and plasmid pTA1. Average read coverage of the plasmid was consistently high (∼2,000×; supplementary fig. S3*A*, Supplementary Material online), whereas average coverage of the *Tatumella* sp. TA1 chromosome was lower (∼500×; supplementary fig. S3*B*, Supplementary Material online). There were some regions of the *Tatumella* sp. TA1 chromosome with high coverage, which are presumably either highly conserved between the *Ca.* E. dacicola and *Tatumella* sp. TA1 chromosomes, or duplicated between the chromosome and the plasmid. For example, the ∼50 kb region (coordinates 700,000–750,000 bp) of the *Tatumella* sp. TA1 chromosome that had the highest mapping coverage of the Oroville reads (6,382×) encodes the Trb operon that is also encoded in the pTA1 plasmid (supplementary fig. S3*B*, Supplementary Material online). On the basis of these analyses, pTA1 may have been horizontally transferred between *Tatumella* sp. TA1 and *Ca*. E. dacicola at some point in their evolutionary history, potentially via another co-occurring member of the gut or environmental microbiota.

Exchanges of genetic material between bacteria are facilitated by extracellular surface structures (Thomas and Nielsen 2005), components of which are encoded in the genomes of the *B. oleae* gut microbiota: Components of the IncF Tra transfer system of conjugative plasmids, encoding F-like pili, are present in the two most complete *Ca.* E. dacicola assemblies, IL and Oroville (supplementary table S4*B* and *C*, Supplementary Material online). In addition, plasmid pTA1 encodes a Trb operon and the four essential components for conjugative transfer: Origin of transfer site (*oriT*), relaxase, type IV coupling protein, and a type IV secretion system (T4SS), in this case a Tra operon (Llosa et al. 2002; De La Cruz et al. 2010), potentiating the exchange of genetic material. The presence of these genes indicates that pTA1 is self-transmissible, in line with its detection in the *Ca.* E. dacicola Oroville assembly.

Some extracellular surface structures that mediate the exchange of genetic material between bacteria, such as T4SS, also facilitate adhesion and molecular exchange with eukaryotic cells (Alvarez-Martinez and Christie 2009). T4SS are diverse; VirB-like T4SS encode short and rigid pili, whereas the F-pili T4SS for conjugative transfer tend to be long and flexible (Clarke et al. 2008). A conserved VirB-like T4SS operon encoding the essential genes for pilus formation and substrate trafficking (VirB1-11 and VirD4, Wallden et al. 2010) was detected in all three *Ca.* E. dacicola assemblies, but was not detected in either *Enterobacter* sp. OLF or *Tatumella* sp. TA1 (supplementary table S4*A*–*E*, Supplementary Material online). There is evidence for expression of the *Ca.* E. dacicola VirB-like T4SS operon during *B. oleae* juvenile development in ripening olives (Pavlidi et al. 2017) and for the presence of pili-like structures in the esophageal bulb (Poinar et al. 1975). Depending on their structure, pili can deliver a range of effector molecules including proteins and nucleic acids to both prokaryotic and eukaryotic cells. T4SS are involved in the transition between motility and sessility, for example in the process of biofilm formation (Christie and Vogel 2000), and can also mediate the delivery of effector proteins directly to the cytoplasm of eukaryotic cells during the process of infection in pathogenic bacteria (Cascales and Christie 2003), and root nodule colonization in symbiotic *Rhizobia* (Deakin and Broughton 2009); these effector proteins can induce regulatory changes in the eukaryotic cell, triggering modification of cellular conditions or conditions in the environment to facilitate growth or invasion (Burns 2003). Therefore, the T4SS system pili in *Ca.* E. dacicola may also play a—yet unknown—role in the *B. oleae*—*Ca.* E. dacicola symbiosis, for example by the establishment of *Ca.* E. dacicola biofilms (Estes et al. 2009). One potential function to explore in future studies might be the coordination of gut lumen colonization and subsequent resource exchange between the host and symbiont.

## Conclusions

In this study, we aimed to gain understanding of the advantages of symbiosis for the olive fruit fly *B. oleae* by integrating various sources of genetic information on its microbial gut community. This led to the identification, by 16S rRNA gene sequencing and culture-dependent methods, of a novel facultative member of the gut microbiota, *Tatumella* sp. TA1, which associates with Mediterranean populations of *B. oleae* throughout the lifecycle. *Tatumella* sp. TA1 is phylogenetically distinct from the US-restricted facultative symbiont *Enterobacter* sp. OLF, highlighting the population-dependent nature of gut microbiota composition in the olive fruit fly. Comparative genomics indicated that the obligate symbiont *Ca.* E. dacicola, as well as *Tatumella* sp. TA1 and *Enterobacter* sp. OLF, which are all stable components of the *B. oleae* microbiota throughout the olive fruit fly lifecycle, encode genes that allow the use of urea as a nitrogen source. The hydrolysis of urea to ammonia is encoded by genes with distinct phylogenetic origins in each organism: HGT of a urease operon in *Ca.* E. dacicola, an endogenous urease operon in *Enterobacter* sp. OLF, and an endogenous alternative enzymatic machinery (urea carboxylase and allophanate hydrolase) in *Tatumella* sp. TA1. These findings provide a potential mechanistic basis for previous experimental evidence of gut microbiota-mediated dietary nitrogen provisioning during adulthood, but emphasize the need for experimental validation of metabolic cross-feeding between the host and obligate symbiont. A hypothesis in this direction, warranting focus in future studies, is that a VirB-like T4SS encoded by *Ca.* E. dacicola, but missing from the genomes of *Enterobacter* sp. OLF and *Tatumella* sp. TA1, may facilitate specific interactions with the host at the symbiotic interface. Besides T4SS, our study further highlighted extracellular surface structures, encoded in the genomes of the obligate and the facultative symbionts, as mediators of DNA transfer: Detection of a plasmid shared by geographically distinct *Ca.* E. dacicola and *Tatumella* sp. TA1, along with the horizontal acquisition of genes important to symbiosis function (urease in this study, genes related to amino-acid degradation in Estes, Hearn, Agrawal, et al. 2018) and the abundance of MGEs in the obligate symbiont genome, indicate that previous and on-going genetic exchanges between gut community members are important determinants of symbiotic interactions with *B. oleae*.

## Supplementary Material

Supplementary data are available at *Genome Biology and Evolution* online.

## Supplementary Material

evz258_Supplementary_Data

## Notes

**Data deposition:** This project has been deposited at GenBank under the accessions PRJNA321173, CPO033727 and CP033728.

## References

[evz258-B1] Altschul SF, Gish W, Miller W, Myers EW, Lipman DJ. 1990 Basic local alignment search tool. J Mol Biol. 215(3):403–410. 2231712 10.1016/S0022-2836(05)80360-2

[evz258-B2] Alvarez-Martinez CE, Christie PJ. 2009 Biological diversity of prokaryotic type IV secretion systems. Microbiol Mol Biol Rev. 73(4):775–808. 19946141 10.1128/MMBR.00023-09 PMC2786583

[evz258-B3] Baker KS, et al 2018 Horizontal antimicrobial resistance transfer drives epidemics of multiple Shigella species. Nat Commun. 9(1):1462. 29654279 10.1038/s41467-018-03949-8 PMC5899146

[evz258-B4] Ben-Yosef M, Aharon Y, Jurkevitch E, Yuval B. 2010 Give us the tools and we will do the job: symbiotic bacteria affect olive fly fitness in a diet-dependent fashion. Proc R Soc B. 277(1687):1545–1552. 10.1098/rspb.2009.2102 PMC2871834 20071385

[evz258-B5] Ben-Yosef M, Pasternak Z, Jurkevitch E, Yuval B. 2014 Symbiotic bacteria enable olive flies (*Bactrocera oleae*) to exploit intractable sources of nitrogen. J Evol Biol. 27(12):2695–2705. 25403559 10.1111/jeb.12527

[evz258-B6] Ben-Yosef M, Pasternak Z, Jurkevitch E, Yuval B. 2015 Symbiotic bacteria enable olive fly larvae to overcome host defences. R Soc Open Sci. 2(7):150170. 26587275 10.1098/rsos.150170 PMC4632588

[evz258-B7] Blow F, Gioti A, et al 2016 Draft genome sequence of the *Bactrocera oleae* symbiont “*Candidatus* Erwinia dacicola.” Genome Announc. 4(5): e00896–16. 10.1128/genomeA.00896-16 PMC5026430 27634990

[evz258-B8] Blow F, Vontas J, Darby AC. 2016 Draft genome sequence of *Stenotrophomonas maltophilia* SBo1 isolated from *Bactrocera oleae*. Genome Announc. 4(5): e00905–16. 10.1128/genomeA.00905-16 PMC5034120 27660769

[evz258-B9] Blow F, Vontas J, Darby AC. 2017 Draft genome sequence of chryseobacterium strain CBo1 isolated from *Bactrocera oleae*. Genome Announc. 5(18): e00177–17. 10.1128/genomeA.00177-17 PMC5477180 28473371

[evz258-B10] Bolger AM, Lohse M, Usadel B. 2014 Trimmomatic: a flexible trimmer for illumina sequence data. Bioinformatics 30(15):2114–2120. 24695404 10.1093/bioinformatics/btu170 PMC4103590

[evz258-B11] Bosco L, Giacometto E, Tavella L. 2008 Colonization and predation of thrips (Thysanoptera: Thripidae) by *Orius* spp. (Heteroptera: Anthocoridae) in sweet pepper greenhouses in Northwest Italy. Biol Control. 44(3):331–340.

[evz258-B304] Brady C, et al. 2010. Description of Gibbsiella quercinecans gen. nov., sp. nov., associated with Acute Oak Decline. Syst. Appl. Microbiol. 33:444–450. 10.1016/j.syapm.2010.08.006 21115313

[evz258-B12] Burns DL. 2003 Type IV transporters of pathogenic bacteria. Curr Opin Microbiol. 6(1):29–34. 12615216 10.1016/s1369-5274(02)00006-1

[evz258-B13] Caporaso JG, Bittinger K, et al 2010 PyNAST: a flexible tool for aligning sequences to a template alignment. Bioinformatics 26(2):266–267. 19914921 10.1093/bioinformatics/btp636 PMC2804299

[evz258-B14] Caporaso JG, Kuczynski J, et al 2010 QIIME allows analysis of high-throughput community sequencing data. Nat Methods. 7(5):335–336. 20383131 10.1038/nmeth.f.303 PMC3156573

[evz258-B15] Caporaso JG, et al 2011 Global patterns of 16S rRNA diversity at a depth of millions of sequences per sample. Proc Natl Acad Sci USA. 108(Suppl 1):4516–4522. 20534432 10.1073/pnas.1000080107 PMC3063599

[evz258-B16] Capuzzo C, Firrao G, Mazzon L, Squartini A, Girolami V. 2005 “*Candidatus* Erwinia dacicola”, a coevolved symbiotic bacterium of the olive fly *Bactrocera oleae* (Gmelin). Int J Syst Evol Microbiol. 55(4):1641–1647. 16014495 10.1099/ijs.0.63653-0

[evz258-B17] Cascales E, Christie PJ. 2003 The versatile bacterial type IV secretion systems. Nat Rev Microbiol. 1(2):137–149. 15035043 10.1038/nrmicro753 PMC3873781

[evz258-B18] Castresana J. 2000 Selection of conserved blocks from multiple alignments for their use in phylogenetic analysis. Mol Biol Evol. 17(4):540–552. 10742046 10.1093/oxfordjournals.molbev.a026334

[evz258-B19] Chandler JA, James PM, Jospin G, Lang JM. 2014 The bacterial communities of *Drosophila suzukii* collected from undamaged cherries. PeerJ 2:e474. 25101226 10.7717/peerj.474 PMC4121540

[evz258-B20] Chen X, et al 2017 Comparative genomics of facultative bacterial symbionts isolated from European *Orius* species reveals an ancestral symbiotic association. Front Microbiol. 8: 1969. 10.3389/fmicb.2017.01969 PMC5641365 29067021

[evz258-B21] Chin C-S, et al 2013 Nonhybrid, finished microbial genome assemblies from long-read SMRT sequencing data. Nat Methods. 10(6):563–569. 23644548 10.1038/nmeth.2474

[evz258-B22] Christie PJ, Vogel JP. 2000 Bacterial type IV secretion: conjugation systems adapted to deliver effector molecules to host cells. Trends Microbiol. 8(8):354–360. 10920394 10.1016/s0966-842x(00)01792-3 PMC4847720

[evz258-B23] Clarke M, Maddera L, Harris RL, Silverman PM. 2008 F-pili dynamics by live-cell imaging. Proc Natl Acad Sci USA. 105(46):17978–17981. 19004777 10.1073/pnas.0806786105 PMC2582581

[evz258-B303] D'Amore R, et al. 2016. A comprehensive benchmarking study of protocols and sequencing platforms for 16S rRNA community profiling. BMC Genomics. 17:55. 10.1186/s12864-015-2194-9 PMC4712552 26763898

[evz258-B24] Deakin WJ, Broughton WJ. 2009 Symbiotic use of pathogenic strategies: rhizobial protein secretion systems. Nat Rev Microbiol. 7(4):312–320. 19270720 10.1038/nrmicro2091

[evz258-B302] Denman S, et al. 2012. Brenneria goodwinii sp. nov., associated with acute oak decline in the UK. *Int J Syst Evol Microbiol.* 62:2451–2456. 10.1099/ijs.0.037879-0 22140177

[evz258-B25] De La Cruz F, Frost LS, Meyer RJ, Zechner EL. 2010 Conjugative DNA metabolism in Gram-negative bacteria. FEMS Microbiol Rev. 34(1):18–40. 19919603 10.1111/j.1574-6976.2009.00195.x

[evz258-B26] DeSantis TZ, et al 2006 Greengenes, a chimera-checked 16S rRNA gene database and workbench compatible with ARB. Appl Environ Microbiol. 72(7):5069–5072. 16820507 10.1128/AEM.03006-05 PMC1489311

[evz258-B27] de Vries EJ, Jacobs G, Breeuwer JA. 2001 Growth and transmission of gut bacteria in the Western flower thrips, Frankliniella occidentalis. J Invertebr Pathol. 77(2):129–137. 11273693 10.1006/jipa.2001.5010

[evz258-B28] de Vries EJ, Jacobs G, Sabelis MW, Menken SBJ, Breeuwer J. 2004 Diet-dependent effects of gut bacteria on their insect host: the symbiosis of *Erwinia* sp. and western flower thrips. Proc R Soc Lond B. 271(1553):2171–2178. 10.1098/rspb.2004.2817 PMC1691834 15475338

[evz258-B29] Dillon RJ, Dillon VM. 2004 The gut bacteria of insects: nonpathogenic interactions. Annu Rev Entomol. 49(1):71–92. 14651457 10.1146/annurev.ento.49.061802.123416

[evz258-B30] Djambazian H, et al. 2018. De novo genome assembly of the olive fruit fly (Bactrocera oleae) developed through a combination of linked-reads and long-read technologies. bioRxiv. 505040. doi: 10.1101/505040. Accessed March 27, 2019.

[evz258-B31] Douglas AE. 2009 The microbial dimension in insect nutritional ecology. Funct Ecol. 23(1):38–47.

[evz258-B32] Edgar RC. 2004 MUSCLE: multiple sequence alignment with high accuracy and high throughput. Nucleic Acids Res. 32(5):1792–1797. 15034147 10.1093/nar/gkh340 PMC390337

[evz258-B33] Edgar RC. 2010 Search and clustering orders of magnitude faster than BLAST. Bioinformatics 26(19):2460–2461. 20709691 10.1093/bioinformatics/btq461

[evz258-B34] Edgar RC, Haas BJ, Clemente JC, Quince C, Knight R. 2011 UCHIME improves sensitivity and speed of chimera detection. Bioinformatics 27(16):2194–2200. 21700674 10.1093/bioinformatics/btr381 PMC3150044

[evz258-B35] Estes AM, Hearn DJ, Bronstein JL, Pierson EA. 2009 The olive fly endosymbiont, “*Candidatus* Erwinia dacicola,” switches from an intracellular existence to an extracellular existence during host insect development. Appl Environ Microbiol. 75(22):7097–7106. 19767463 10.1128/AEM.00778-09 PMC2786516

[evz258-B36] Estes AM, Hearn DJ, Burrack HJ, Rempoulakis P, Pierson EA. 2012 Prevalence of *Candidatus* Erwinia dacicola in wild and laboratory olive fruit fly populations and across developmental stages. Environ Entom. 41(2):265–274. 10.1603/EN11245 22506998

[evz258-B37] Estes AM, Hearn DJ, Agrawal S, Pierson EA, Dunning Hotopp JC. 2018 Comparative genomics of the Erwinia and Enterobacter olive fly endosymbionts. Sci Rep. 8(1):15936. 30374192 10.1038/s41598-018-33809-w PMC6205999

[evz258-B38] Estes AM, Hearn DJ, Nadendla S, Pierson EA, Dunning Hotopp JC. 2018a Draft genome sequence of *Enterobacter* sp. Microbiol Resour Announc. 7: e01068–18. 10.1128/MRA.01068-18 PMC6256528 30533936

[evz258-B39] Estes AM, Hearn DJ, Nadendla S, Pierson EA, Dunning Hotopp JC. 2018b Draft genome sequence of erwinia dacicola, a dominant endosymbiont of olive flies. Microbiol Res Announc. 7:e01067–18. 10.1128/MRA.01067-18 PMC6256602 30533624

[evz258-B40] Estes AM, Segura DF, Jessup A, Wornoayporn V, Pierson EA. 2014 Effect of the symbiont *Candidatus* Erwinia dacicola on mating success of the olive fly *Bactrocera oleae* (Diptera: tephritidae). Int J Trop Insect Sci. 34(S1):S123–S131.

[evz258-B41] Facey PD, et al 2015 Draft genomes, phylogenetic reconstruction, and comparative genomics of two novel cohabiting bacterial symbionts isolated from Frankliniella occidentalis. Genome Biol Evol. 7(8):2188–2202. 26185096 10.1093/gbe/evv136 PMC4558854

[evz258-B42] Feldhaar H, et al 2007 Nutritional upgrading for omnivorous carpenter ants by the endosymbiont Blochmannia. BMC Biol. 5(1):48. 17971224 10.1186/1741-7007-5-48 PMC2206011

[evz258-B43] Gil R, et al 2008 Massive presence of insertion sequences in the genome of SOPE, the primary endosymbiont of the rice weevil *Sitophilus oryzae*. Int Microbiol. 11(1):41–48. 18683631

[evz258-B44] Hacker J, Kaper JB. 2000 Pathogenicity islands and the evolution of microbes. Annu Rev Microbiol. 54(1):641–679. 11018140 10.1146/annurev.micro.54.1.641

[evz258-B45] Hollis DG, et al 1981 *Tatumella ptyseos* gen. nov., sp. nov., a member of the family Enterobacteriaceae found in clinical specimens. J Clin Microbiol. 14(1):79–88. 7263854 10.1128/jcm.14.1.79-88.1981 PMC271905

[evz258-B46] Jørgensen TS, Kiil AS, Hansen MA, Sørensen SJ, Hansen LH. 2014 Current strategies for mobilome research. Front Microbiol. 5:750. 25657641 10.3389/fmicb.2014.00750 PMC4302988

[evz258-B47] Joshi N, Fass JN. 2011 Sickle-A windowed adaptive trimming tool for FASTQ files using quality. Online publication. https://github.com/najoshi/sickle

[evz258-B48] Juhas M, et al 2009 Genomic islands: tools of bacterial horizontal gene transfer and evolution. FEMS Microbiol Rev. 33(2):376–393. 19178566 10.1111/j.1574-6976.2008.00136.x PMC2704930

[evz258-B49] Kashima T, Nakamura T, Tojo S. 2006 Uric acid recycling in the shield bug, *Parastrachia japonensis* (Hemiptera: Parastrachiidae), during diapause. J Insect Physiol. 52(8):816–825. 16797581 10.1016/j.jinsphys.2006.05.003

[evz258-B50] Koskinioti P, et al 2019 The effects of geographic origin and antibiotic treatment on the gut symbiotic communities of *Bactrocera oleae* populations. Entomol Exp Appl. 167(3):197–208.

[evz258-B51] Kounatidis I, et al 2009 Acetobacter tropicalis is a major symbiont of the olive fruit fly (*Bactrocera oleae*). Appl Environ Microbiol. 75(10):3281–3288. 19304818 10.1128/AEM.02933-08 PMC2681620

[evz258-B52] Langmead B, Trapnell C, Pop M, Salzberg SL. 2009 Ultrafast and memory-efficient alignment of short DNA sequences to the human genome. Genome Biol. 10(3):R25. 19261174 10.1186/gb-2009-10-3-r25 PMC2690996

[evz258-B53] Li H, et al 2009 The sequence alignment/map format and SAMtools. Bioinformatics 25(16):2078–2079. 19505943 10.1093/bioinformatics/btp352 PMC2723002

[evz258-B54] Li L, Stoeckert CJ Jr, Roos DS. 2003 OrthoMCL: identification of ortholog groups for eukaryotic genomes. Genome Res. 13(9):2178–2189. 12952885 10.1101/gr.1224503 PMC403725

[evz258-B55] Llosa M, Gomis-Rüth FX, Coll M, de la Cruz F. 2002 Bacterial conjugation: a two-step mechanism for DNA transport. Mol Microbiol. 45(1):e00177–17. 10.1046/j.1365-2958.2002.03014.x 12100543

[evz258-B56] Marín-Cevada V, et al 2010 *Tatumella ptyseos*, an unrevealed causative agent of pink disease in pineapple. J Phytopathol. 158:93–99.

[evz258-B57] Martin M. 2011 Cutadapt removes adapter sequences from high-throughput sequencing reads. EMBnet J. 17(1):10.

[evz258-B58] Masella AP, Bartram AK, Truszkowski JM, Brown DG, Neufeld JD. 2012 PANDAseq: paired-end assembler for illumina sequences. BMC Bioinformatics 13(1):31. 22333067 10.1186/1471-2105-13-31 PMC3471323

[evz258-B301] McCutcheon JP, Moran NA. 2011. Extreme genome reduction in symbiotic bacteria. Nat. Rev. Microbiol. 10:13–26. 10.1038/nrmicro2670 22064560

[evz258-B59] Minh BQ, Nguyen MAT, von Haeseler A. 2013 Ultrafast approximation for phylogenetic bootstrap. Mol Biol Evol. 30(5):1188–1195. 23418397 10.1093/molbev/mst024 PMC3670741

[evz258-B60] Mouden S, Sarmiento KF, Klinkhamer PG, Leiss KA. 2017 Integrated pest management in western flower thrips: past, present and future. Pest Manag Sci. 73(5):813–822. 28127901 10.1002/ps.4531 PMC5396260

[evz258-B61] Nguyen L-T, Schmidt HA, von Haeseler A, Minh BQ. 2015 IQ-TREE: a fast and effective stochastic algorithm for estimating maximum-likelihood phylogenies. Mol Biol Evol. 32(1):268–274. 25371430 10.1093/molbev/msu300 PMC4271533

[evz258-B62] Nikolenko SI, Korobeynikov AI, Alekseyev MA. 2013 BayesHammer: Bayesian clustering for error correction in single-cell sequencing. BMC Genomics 14(Suppl 1):S7. 10.1186/1471-2164-14-S1-S7 PMC3549815 23368723

[evz258-B63] Overbeek R, et al 2014 The SEED and the Rapid Annotation of microbial genomes using Subsystems Technology (RAST). Nucl Acids Res. 42(D1):D206–D214. 24293654 10.1093/nar/gkt1226 PMC3965101

[evz258-B64] Palmer M, et al 2017 Phylogenomic resolution of the bacterial genus Pantoea and its relationship with *Erwinia* and *Tatumella*. Antonie Van Leeuwenhoek. 110(10):1287–1309. 28255640 10.1007/s10482-017-0852-4

[evz258-B65] Pavlidi N, et al 2017 Transcriptomic responses of the olive fruit fly *Bactrocera oleae* and its symbiont *Candidatus* Erwinia dacicola to olive feeding. Sci Rep. 7:42633. 28225009 10.1038/srep42633 PMC5320501

[evz258-B66] Petersen TN, Brunak S, von Heijne G, Nielsen H. 2011 SignalP 4.0: discriminating signal peptides from transmembrane regions. Nat Methods. 8(10):785–786. 21959131 10.1038/nmeth.1701

[evz258-B67] Petridis M, Bagdasarian M, Waldor MK, Walker E. 2006 Horizontal transfer of Shiga toxin and antibiotic resistance genes among *Escherichia coli* strains in house fly (Diptera: Muscidae) gut. J Med Entomol. 43(2):288–295. 16619613 10.1603/0022-2585(2006)043[0288:htosta]2.0.co;2

[evz258-B68] Poinar GO Jr, Hess RT, Tsitsipis JA. 1975 Ultrastructure of the bacterial symbiotes in the pharyngeal diverticulum of *Dacus oleae* (Gmelin) (Trypetidae; Diptera). Acta Zool. 56(1):77–84.

[evz258-B69] Potrikus CJ, Breznak JA. 1981 Gut bacteria recycle uric acid nitrogen in termites: a strategy for nutrient conservation. Proc Natl Acad Sci U S A. 78(7):4601–4605. 16593064 10.1073/pnas.78.7.4601 PMC319841

[evz258-B70] Quast C, et al 2012 The SILVA ribosomal RNA gene database project: improved data processing and web-based tools. Nucleic Acids Res. 41(D1):D590–D596. 23193283 10.1093/nar/gks1219 PMC3531112

[evz258-B71] R Core Team 2017 R: a language and environment for statistical computing. Vienna (Austria): R Foundation for Statistical Computing https://www.R-project.org/.

[evz258-B72] Ran L, et al 2010 Genome erosion in a nitrogen-fixing vertically transmitted endosymbiotic multicellular cyanobacterium. PLoS One 5(7):e11486. 20628610 10.1371/journal.pone.0011486 PMC2900214

[evz258-B73] Rinke C, et al 2013 Insights into the phylogeny and coding potential of microbial dark matter. Nature 499(7459):431–437. 23851394 10.1038/nature12352

[evz258-B74] Sabree ZL, Kambhampati S, Moran NA. 2009 Nitrogen recycling and nutritional provisioning by Blattabacterium, the cockroach endosymbiont. Proc Natl Acad Sci U S A. 106(46):19521–19526. 19880743 10.1073/pnas.0907504106 PMC2780778

[evz258-B75] Sacchetti P, et al 2008 Relationships between the olive fly and bacteria. J Appl Entomol. 132(9–10):682–689.

[evz258-B76] Salem H, Florez L, Gerardo N, Kaltenpoth M. 2015 An out-of-body experience: the extracellular dimension for the transmission of mutualistic bacteria in insects. Proc R Soc B. 282(1804):20142957. 10.1098/rspb.2014.2957 PMC4375872 25740892

[evz258-B77] Sasaki T, Kawamura M, Ishikawa H. 1996 Nitrogen recycling in the brown planthopper, *Nilaparvata lugens*: involvement of yeast-like endosymbionts in uric acid metabolism. J Insect Physiol. 42(2):125–129.

[evz258-B78] Seemann T. 2014 Prokka: rapid prokaryotic genome annotation. Bioinformatics 30(14):2068–2069.24642063

[evz258-B79] Smillie CS, et al 2011 Ecology drives a global network of gene exchange connecting the human microbiome. Nature 480(7376):241–244. 22037308 10.1038/nature10571

[evz258-B80] Snyder AK, McMillen CM, Wallenhorst P, Rio RV. 2011 The phylogeny of Sodalis-like symbionts as reconstructed using surface-encoding loci. FEMS Microbiol Lett. 317(2):143–151. 21251054 10.1111/j.1574-6968.2011.02221.x PMC3064736

[evz258-B81] Soler-Rivas C, Espin JC, Wichers HJ. 2000 Oleuropein and related compounds. J Sci Food Agric. 80(7):1013–1023.

[evz258-B82] Stecher B, et al 2012 Gut inflammation can boost horizontal gene transfer between pathogenic and commensal Enterobacteriaceae. Proc Natl Acad Sci U S A. 109(4):1269–1274. 22232693 10.1073/pnas.1113246109 PMC3268327

[evz258-B83] Syed MA, Lévesque CM. 2012 Chromosomal bacterial type II toxin–antitoxin systems. Can J Microbiol. 58(5):553–562. 22506850 10.1139/w2012-025

[evz258-B84] Thomas CM, Nielsen KM. 2005 Mechanisms of, and barriers to, horizontal gene transfer between bacteria. Nat Rev Microbiol. 3(9):711–721. 16138099 10.1038/nrmicro1234

[evz258-B85] Tzanakakis M. 2003 Seasonal development and dormancy of insects and mites feeding on olive: a review. Neth J Zool. 52(2):87–224.

[evz258-B86] Wallden K, Rivera-Calzada A, Waksman G. 2010 Microreview: type IV secretion systems: versatility and diversity in function. Cell Microbiol. 12(9):1203–1212. 20642798 10.1111/j.1462-5822.2010.01499.x PMC3070162

[evz258-B87] Wilkins B, Lanka E. 1993 DNA processing and replication during plasmid transfer between gram-negative bacteria In. Clewell DB, editor. Bacterial conjugation. Boston (MA): Springer US p. 105–136.

[evz258-B88] Zhang C, Rabiee M, Sayyari E, Mirarab S. 2018 ASTRAL-III: polynomial time species tree reconstruction from partially resolved gene trees. BMC Bioinformatics 19(S6):153. 29745866 10.1186/s12859-018-2129-y PMC5998893

