# Supplementary material for: Functional Genomics of a Symbiotic Community: Shared Traits in the Olive Fruit Fly Gut Microbiota
Source: Genome Biol Evol. 2019 Dec 12;12(2):3778–91. doi: 10.1093/gbe/evz258 (PMC6999849; doi:10.1093/gbe/evz258)
Supplement: evz258_Supplementary_Data [file evz258_supplementary_data.pdf]

Supplementary material available via figshare links:

Figure S1. Hypothesized microbial nitrogen recycling pathways from host waste products. UO (Uricase E.C. 1.7.3.3); ALN (Allantoinase E.C. 3.5.2.5); ALC (Allantoicase E.C. 3.5.3.4); UL (Ureidoglycolate lyase E.C. 4.3.2.3); URE (Urease 3.5.1.5); GS (Glutamine synthetase E.C. 6.3.1.2).
https://doi.org/10.6084/m9.figshare.10029050

Figure S2. Mauve whole genome alignment of the pTA1 plasmid assembly from *Tatumella* sp. TA1 isolated from Mediterranean populations of *B. oleae* (*Tatumella* sp. TA1) and *Ca.* E. dacicola sequenced from US populations of *B. oleae* (Oroville).
https://doi.org/10.6084/m9.figshare.10029077

Figure S3. Coverage of *Ca.* E. dacicola Oroville reads that mapped to the *Tatumella* sp. TA1 (*A*) pTA1 plasmid and (*B*) chromosome.
https://doi.org/10.6084/m9.figshare.10029101

Supplementary tables figshare link:
https://doi.org/10.6084/m9.figshare.8044556

Table S1. Functional annotations and corresponding sequences for the genomes of *(A-C) Ca.* E. dacicola – three available assemblies, *(D) Tatumella* sp. TA1 and *(E) Enterobacter* sp. OLF, as well as for *(F)* the transcriptome of *Ca.* E. dacicola (ribosomal RNAs excluded). The annotations come from RAST subsystems.

Table S2. GenBank accession numbers and metadata for sequences used in phylogenetic analyses. (*A*) Whole genome sequences used for ortholog picking and phylogenetic reconstruction of the species and reference trees (*B*) Amino acid sequences used for the *ureC* gene tree.

Table S3. Presence and expression of genes related to nitrogen assimilation in *Ca.* E. dacicola and *Tatumella* sp. TA1. *(A)* Urea hydrolysis and nitrogen recycling pathways *(B)* Amino acid biosynthetic pathways.

Table S4. Functional annotation of *(A-C) Ca.* E. dacicola - three available assemblies, *(D) Tatumella* sp. TA1 and *(E) Enterobacter* sp. OLF genomes for extracellular surface structure components, by assignment of genes to the KEGG category “Membrane Transport” using RAST subsystems.
